# Time series analysis of survival and oviposition cycle duration of *Anopheles funestus* (Giles) in Mozambique

**DOI:** 10.7717/peerj.15230

**Published:** 2023-05-29

**Authors:** Jacques D. Charlwood, Thomas A. Smith, Ayubo Kampango, Erzelia V. E. Tomas, Nakul Chitnis

**Affiliations:** 1 DBL Centre for Health Research and Development, Department for Veterinary Pathobiology, Faculty of Life Sciences, University of Copenhagen, Copenhagen, Denmark; 2 Mozambican-Danish Rural Malaria Initiative (MOZDAN), Morrumbene, Inhambane Province, Mozambique; 3 Global Health and Tropical Medicine, Instituto de Higiene e Medicina Tropical, Lisbon, Portugal; 4 Department of Epidemiology and Public Health, Swiss Tropical and Public Health Institute, Allschwil, Switzerland; 5 University of Basel, Basel, Switzerland; 6 Sector de Estudo de Vectores, Instituto Nacional de Saúde, Vila de Marracuene, Província de Maputo, Mozambique; 7 Department of Zoology and Entomology, University of Pretoria, Hatfield, South Africa

**Keywords:** Entomology, *Anopheles funestus*, Survival, Oviposition cycle duration, Feeding cycle duration, Statistics, Time series analysis, Mozambique, Mosquito, Feeding cycle model

## Abstract

**Background:**

Survival and gonotrophic cycle duration are important determinants of the vectorial capacity of malaria vectors but there are a limited number of approaches to estimate these quantities from field data. Time-series of observations of mosquitoes at different stages in the life-cycle are under-used.

**Methods:**

*Anopheles funestus* mosquitoes were caught using various methods over a 7.6-year period in Furvela, Mozambique. Survival and oviposition cycle duration were estimated using (i) an existing time-series approach for analysing dissections of mosquitoes caught in light-traps, extended to allow for variability in the duration of the cycle; (ii) an established approach for estimating cycle duration from resting collection data; (iii) a novel time-series approach fitted to numbers and categories of mosquitoes caught in exit-traps.

**Results:**

Data were available from 7,396, 6,041 and 1,527 trap-nights for exit-traps, light-traps and resting collections respectively. Estimates of cycle duration varied considerably between the different methods. The estimated proportion of female mosquitoes surviving each day of 0.740 (95% credible interval [0.650–0.815]) derived from light-trap data was much lower than the estimated daily survival of male mosquitoes from the model fitted to exit-trap data (0.881, 95% credible interval [0.747–0.987]). There was no tendency for the oviposition cycle to become shorter at higher temperature while the odds of survival of females through the cycle was estimated to be multiplied by 1.021 for every degree of mean weekly temperature increase (95% credible interval [0.991–1.051]). There was negligible temperature dependence and little inter-annual variation in male survival.

**Discussion:**

The time-series approach fitted to the exit-traps suggests that male *An. funestus* have higher survival than do females, and that male survival was temperature independent and unaffected by the introduction of long-lasting insecticidal nets (LLINs). The patterns of temperature dependence in females are at variance with results of laboratory studies. Time series approaches have the advantage for estimating survival that they do not depend on representative sampling of mosquitoes over the whole year. However, the estimates of oviposition cycle duration were associated with considerable uncertainty, which appears to be due to variability between insects in the duration of the resting period, and the estimates based on exit-trap data are sensitive to assumptions about relative trapping efficiencies.

## Introduction

*Anopheles funestus* is a major vector of malaria in southern Africa (Sinka et al., 2010; Wiebe et al., 2017), but until recently it was considered amenable to control by indoor residual spraying (IRS), because males, newly emerged virgin females, and older females at all stages of gonotrophic development rest inside houses. The rapid spread of metabolic resistance to pyrethroids from South Africa to Mali (including Mozambique) in this species (Knox et al., 2014) has meant that it has become the major vector in Mozambique and southern Tanzania.

The Funestus group was first designated by Gillies & DeMeillon (1968) to describe a set of closely related species that showed small morphological differences (such as the presence or absence of a fringe spot of light scales at the distal end of vein 5.2 on the wing). Larvae are found in permanent or semi-permanent sites with emergent or floating vegetation. It has an optimum development temperature of 25 °C and develops less quickly when exposed to fluctuating rather than stable temperatures (Lyons, Coetzee & Chown, 2013). Since the work of Jepson, Moutia & Courtois (1947) the chief factors controlling *An. funestus* breeding under natural conditions have been thought to be temperature and food supply. A recent field survey in Tanzania found the optimum temperature for breeding to range from 25.2 °C to 28.8 °C (Nambunga et al., 2020).

Increasing mosquito mortality is generally the most immediate effect of interventions against adult malaria vectors, but measurement of Anopheline survival in the field is problematic. Assessment of parous rates (*i.e*., the proportion of insects that have laid eggs) by dissection remains the standard method and this can be used as an estimate of survival per oviposition cycle. To derive the epidemiologically important quantity of survival per day, estimates of the duration of the oviposition cycle are also needed, but these are rarely available from the field. Oviposition cycle duration in *An. funestus* was estimated to be 3 days irrespective of season by Gillies & Wilkes (1963) who analysed and dissected mosquitoes from pyrethrum spray collections of individuals caught resting inside houses. They found that above 26.5 °C gonotrophic development took 2 days but that the insects delayed returning to feed for a day, whilst at lower temperatures, egg development took 3 days but the insects returned to feed immediately after oviposition. They also found a high proportion of pre-gravid insects in their samples in contrast to collections made in West Africa (Gillies & Wilkes, 1963). In West Africa *An. funestus* consists of two genetically distinct lines, only one of which occurs in East Africa (Costantini et al., 1999). The differences in pre-gravid rates may, therefore, reflect the local environment or they may be a genetically based difference.

Capture-recapture (aka mark-release-recapture, MRR) can also provide information on survival rate and the duration of the gonotrophic cycle. Several spectroscopic approaches for age-determination of mosquitoes are also in development (Lambert et al., 2018; Wagner et al., 2023), but none have so far proven practicable as high-throughput alternatives to analysis of relative frequencies of different age-classes of insects.

Mosquito age data from dissection provide valid direct estimates of survival when representative samples of host-seeking mosquitoes are analysed from a full annual cycle including seasonality (Clements & Paterson, 1981) thus representing an average over a full year. When data are only available for part of the year, so that the population is either increasing or decreasing, estimates based only on mosquito age data such as parous rates can be strongly biased (see Supplemental Information). This limits the value of such methods for studying spatial or temporal variation in survival.

Time-series of observations of mosquitoes at different stages in the life-cycle (Birley & Rajagopalan, 1981; Holmes & Birley, 1987) can provide estimates of both survival and cycle duration without the need for a complete annual cycle to be sampled. They can also be used to explore the variability in them over time and in space, though this possibility has rarely been exploited. The original model has been extended to allow for a pre-gravid phase (Mutero & Birley, 1989) and for variation in cycle duration (Birley & Boorman, 1982). In the present publication, Markov chain Monte Carlo (MCMC) methods are used to fit the model with variation in cycle duration, which provides a more robust analysis of uncertainty, and allows fitting to unbalanced datasets with missing data. The data analysed are numbers of *Anopheles funestus* from resting catches, light-traps, and exit-traps in the village of Furvela, Inhambane Province, Mozambique (Charlwood, 2017; Charlwood et al., 2015). A new approach is presented for estimating daily survival using resting catch data, and novel time-series models are developed for analysis of light- and exit-trap data, of female mosquitoes, and for providing estimates of the survival of male mosquitoes.

## Methods

### Study site

The village of Furvela has been described in previous publications of the project (Charlwood, 2011, 2017; Charlwood & Braganca, 2012; Charlwood et al., 2021; Kampango et al., 2013). This is a subsistence farming community, with the main crops being maize, manioc, peanuts, and beans. The single rainy season extends from October to March, with approximately 1,200 mm of rain. Daily mean temperatures, recorded at the nearby site of Vilanculos vary between 18 °C (July) and 30 °C (December) (Charlwood, 2017).

*Plasmodium falciparum* malaria was highly endemic with infection rates about 80% in infants at the health post (Charlwood et al., 2021). Long-lasting insecticide treated mosquito nets (LLINs) were provided to 150 houses closest to the river valley in July 2007. *Anopheles funestus* was the primary vector in the village although three members of the *An. gambiae* complex were also present, especially at the start of the study (Charlwood, 2017).

### Mosquito sampling

Mosquito collections began in June 2001 and continued until January 2009. Mosquitoes were caught in exit-traps, CDC light-traps, resting collections. Details of the trapping methods were described previously (Charlwood, 2017). The first 2 years of light-traps were based on a random set of houses. The later samples were a convenience sample and over-sampled the central parts of the village. Resting (and tent-trap) collections were undertaken on an *ad hoc* basis.

### Field and laboratory techniques

Collected mosquitoes were separated into species or species group according to the keys of Gillies & DeMeillon (1968) and Gillies & Coetzee (1987). Females caught in exit-traps and resting collections were classified according to their abdominal appearance as being ‘unfed’, ‘part-fed’, ‘fully-fed/semi-gravid’ or ‘gravid’, according to the condition of the ovaries.

Dissections were undertaken on unfed mosquitoes collected from the light-traps. Parous status and the presence of ovariolar sacs were determined as described by Charlwood et al. (2003b) and Charlwood et al. (2018).

### Estimates of survival and cycle duration based on aggregated data

If a whole annual seasonal cycle is representatively sampled, then the proportions of mosquitoes in different categories of parity, gravidity, and the presence of ovariolar sacs provide consistent and unbiased estimates of survival and cycle duration. Estimates made using this approach as follows:

${{ P}_{o}}$, *the survival of adult female mosquitoes per oviposition cycle* was estimated by $P_o^{\left( 1 \right)}$, the proportion of host-seeking female mosquitoes in light-traps that are parous (Davidson, 1954).

${\theta _r},\;$ *the duration of the resting period* was estimated from resting catch data using an extension of the approach used by Kulkarni et al. (2006) and Tchuinkam et al. (2010). The proportion of resting mosquitoes recorded as fed (*i.e*., having fed the previous night and still digesting the blood meal) among those that are either fed, semi-gravid, or gravid was estimated by:

(1) $$\matrix{ {{f^{\left( 1 \right)}} = \mathop \sum \limits_t {F}_{{t}}^{\left( {R} \right)}/\mathop \sum \limits_t \left( {{F}_{{t}}^{\left( {R} \right)} + {G}_{{t}}^{\left( {R} \right)}} \right) } \cr }$$

where $t$ represents the trap night and ${F}_{{t}}^{\left( {R} \right)}$ and ${G}_{{t}}^{\left( {R} \right)}$ represent the number of fed and semi-gravid/gravid mosquitoes caught in resting catches, respectively (Table 1). Neglecting mortality while resting, the duration of the resting period is then estimated by $\theta _r^{\left( 1 \right)} = \displaystyle{1 \over {{f^{\left( 1 \right)}}}}$. Kulkarni et al. (2006) based on the assumption of negligible resting stage mortality.

**Table 1:** Notation for time-dependent quantities.

Symbol	Meaning	Trap type	Equation
Directly observed quantities			
${G}_{{t}}^{\left( {R} \right)}$	Total number of gravid or semi-gravid mosquitoes in resting collections	Resting	(1)
${F}_{{t}}^{\left( {R} \right)}$	Total number of fed mosquitoes in resting collections	Resting	(1)
${n}_{{t}}^{\left( {L} \right)}$	Number of light-traps operated	Light	(4)
${T}_{{t}}^{\left( {L} \right)}$	Total number of female mosquitoes caught in light-traps	Light	(2)
${M}_{{t}}^{\left( {L} \right)}$	Total number of parous mosquitoes caught in light-traps	Light	(3)
${D}_{{t}}^{\left( {L} \right)}$	Total number caught in light-traps and dissected	Light	(4)
${n}_{{t}}^{\left( {E} \right)}$	Number of exit-traps operated	Exit	(16)
${G}_{{t}}^{\left( {E} \right)}$	Total number of gravid mosquitoes caught in exit-traps	Exit	(9)
${V}_{{t}}^{\left( {E} \right)}$	Total number of male mosquitoes caught in exit-traps	Exit	(16)
Quantities estimated indirectly			
${m}_{{t}}^{\left( {L} \right)}$	Expected mean number of parous mosquitoes per trap	Light	(2)
${\lambda }_{\rm t}^{\left( {\rm L} \right)}$	Emergence rate as measured by expected number of nulliparous females per light-trap	Light	(6)
${u}_{{t}}^{\left( {E} \right)}$	Expected mean number of unfed or part-fed mosquitoes per trap	Exit	(11)
${g}_{{t}}^{\left( {E} \right)}$	Expected mean number of gravid mosquitoes per trap	Exit	(11)
${v}_{{t}}^{\left( {E} \right)}$	Expected mean number of male mosquitoes per trap	Exit	(15)
${\lambda }_{{t}}^{\left( {\rm E} \right)}$	Emergence rate as measured by expected number of unfed nulliparous females per exit-trap	Exit	(13)

**Note:**

Each quantity is specific for the distinct night $t$.

${a_0}$, *the proportion of host-seeking mosquitoes that return on the same night as oviposition*.

This was estimated by the sac rate, as described by Charlwood et al. (1985) and applied also by Charlwood et al. (1995, 2003a, 2003b, 1997), Molez, Desenfant & Jacques (1998).

${\theta _o}$, *the duration of the full oviposition cycle in days*.

This was calculated as a function of the resting period, ${\theta _r}$ and of ${a_0}$ (Charlwood et al., 2016):

(2) $$\matrix{ {{\theta _o} = {\theta _r} + \left( {1 - {a_0}} \right)/{a_0}} \cr }$$

Substitution of ${\theta _r} = \theta _r^{\left( 1 \right)}$, gave an estimate $\theta _o^{\left( 1 \right)}$ for ${\theta _o}$.

${\rm p}$, *the daily survival of female mosquitoes*

Assuming an exponential survival distribution, daily survival, *p*, is related to ${{\rm P}_{\rm o}}$ by:

(3) $$\matrix{ {\ln p = \displaystyle{{\ln \left( {{{\rm P}_{\rm o}}} \right)} \over {{\theta _o}}}} \cr }$$

Substitution of $\; {\theta _o} = \theta _o^{\left( 1 \right)}$, and ${P_o} = P_o^{\left( 1 \right)}$ gave an estimate ${p^{\left( 1 \right)}}$ for *p*.

#### Allowance for resting stage mortality

If mosquitoes die during the resting stage, with constant daily survival *p*, $f$ is expected to be:

(4) $$\matrix{ {{f^{\left( 2 \right)}} = 1/\mathop \sum \limits_{\tau = 1}^{{\theta _r}} {p^{\tau - 1}} = \displaystyle{{1 - p} \over {1 - {p^{{\theta _r}}}}}.} \cr }$$

Substituting Eqs. (2) and (3) into this gives $f$ as a function of ${{\rm P}_{\rm o}}$ and ${\theta _o}$:

(5) $$\matrix{ {{f^{\left( 2 \right)}} = \displaystyle{{1 - P_o^{1/{\theta _o}}} \over {1 - P_o^{\left( {{\theta _o} - \left( {1 - {a_0}} \right)/{a_0}} \right)/{\theta _o}}}}.} \cr }$$

The observed value of ${f^{\left( 2 \right)}}$, and the estimate $P_o^{\left( 1 \right)}$ for ${{\rm P}_{\rm o}}$ were substituted into Eq. (5). The R uniroot solver was then used to obtain an estimate $\theta _o^{\left( 2 \right)}$ for ${\theta _o}$ that allows for resting-stage mortality. Substitution of this into Eq. (2) gave a corresponding estimate, $\theta _r^{\left( 2 \right)}$, for ${\theta _r}$, and by substituting this into Eq. (3) a value of ${p^{\left( 2 \right)}}$ for $p$.

### Estimates of survival and cycle duration based on time-series analysis

The notation used for time-dependent quantities including the counts of numbers of mosquitoes in different categories is given in Table 1. Upper case Latin letters are used for total numbers of mosquitoes in any category caught in all traps on night *t*. Lower-case letters are used for average numbers per trap-night.

#### Estimation of survival and the duration of the oviposition cycle from light-trap data

Following Birley & Rajagopalan (1981), a further estimate of survival per cycle, $P_o^{\left( 2 \right)}$, can be obtained from short time series of parity data without the need to sample the entire annual cycle, using the relationship:

(6) $$\matrix{ {{m_t} = \displaystyle{{P_o^{\left( 2 \right)}T_{t - {\theta _o}}^{\left( L \right)}} \over {n_{t - {\theta _o}}^{\left( {\rm L} \right)}}},} \cr }$$

where ${m_t}$ is the expected mean number of parous mosquitoes caught in a light-trap on night *t*; ${T_t}$ is the total number of any stage of female mosquitoes; $n_t^{\left( {\rm L} \right)}$ is the number of light-traps operated; and ${\theta _o}$ is the duration of the oviposition cycle in days. Using the Furvela data, a Bayesian extension of this approach was implemented, including allowance for missing data, for variation in the numbers of traps per night, and for variation within the mosquito population in the duration of the cycle (so that $\theta _o^{\left( 3 \right)}$, the estimate of ${\theta _o}$ is a distribution, rather than a single value integer).

In this model the total number of parous *Anopheles funestus* mosquitoes caught in light-traps on night *t* is assumed to be negatively binomially distributed about its expectation:

(7) $$\matrix{ {M_t^{\left( L \right)}\sim {\rm negbin}\left( {E\left( {M_t^{\left( L \right)}} \right),{r^{\left( 1 \right)}}} \right),} \cr }$$

where ${r^{\left( 1 \right)}}$ is a negative binomial dispersion parameter, and the expectation is:

(8) $$\matrix{ {E\left( {M_t^{\left( L \right)}} \right) = \; \displaystyle{{m_t^{\left( L \right)}n_t^{\left( L \right)}D_t^{\left( L \right)}} \over {T_t^{\left( L \right)}}},} \cr }$$

with ${D_t}$ the total number of mosquitoes dissected. The cycle duration, $\theta _o^{\left( 3 \right)},$ is modelled with a normal kernel (truncated to be between 1.5 and 4.5 nights), so that:

(9) $$\matrix{ {m_t^{\left( L \right)} = P_o^{\left( 2 \right)}\mathop \sum \limits_{\tau = 2}^{\tau = 4} \Phi _\tau ^{\left( L \right)}T_{t - \tau }^{\left( L \right)}/n_{t - \tau }^{\left( L \right)}} \cr }$$

where $\tau$ is the lag in days and ${\Phi }_\tau ^{\left( L \right)}$ is the slice of the normal kernel (of mean $\theta _o^{\left( 3 \right)}$ and variance $\sigma _o^{\left( L \right)2}$) assigned to $\tau$, so that ${\Phi }_\tau ^{\left( L \right)} = \Pr \left( {\tau - 0.5 < \theta _o^{\left( 3 \right)} < \; \tau + 0.5} \right)$. ${\theta _{\rm o}}$ takes real values, but the model is in discrete time, with one-day time steps. Effectively this means that a Poisson mixture of discrete values is assumed for $\theta _o^{\left( 3 \right)}$ (this is broadly supported by published distributions for cycle durations (Beier, 1996)).

$\theta _o^{\left( 3 \right)}$, $\sigma _o^{\left( L \right)}$, and $P_o^{\left( 2 \right)}$ were all estimated using an MCMC algorithm in rjags. This required specification of a uniform (1,5) prior for the estimate of $\theta _o^{\left( 3 \right)}$, a uniform (0,1) prior for $P_o^{\left( 2 \right)}$ and gamma priors for $\displaystyle{1 \over {\sigma _o^{\left( L \right)2}}}$ and the dispersion parameter ${r^{\left( 1 \right)}}$. Using Eq. (2), a further estimate of the duration of the resting period, $\theta _r^{\left( 3 \right)}$, was also obtained from $\theta _o^{\left( 3 \right)}$ and ${a_0}$. Substituting the estimates $P_o^{\left( 2 \right)}$ and $\theta _o^{\left( 3 \right)}\;$ into Eq. (3) gave a value of ${p^{\left( 3 \right)}}$ for $p$.

#### Estimation of emergence rates from light-trap data

Noting that the term ‘rate’ is used here to refer to an average number of mosquitoes per trap-night, the population average emergence rate, scaled to correspond to the average light-trap catch was estimated as the number of nulliparous mosquitoes per trap-night, *i.e*.,:

(10) $$\matrix{ { \lambda _t^{\left( {\rm L} \right)} = T_t^{\left( L \right)}/n_t^{\left( {\rm L} \right)} - {m_t}} \cr }$$

For the 217/513 nights in the sampling period for which the value for $T_t^{\left( L \right)}$ was missing, $T_t^{\left( L \right)}$ (and hence $\lambda _t^{\left( {\rm L} \right)}$) was imputed by assigning a log-normal prior to the inter-night variation in light-trap collections, using the observed values for the other nights to determine the mean and variance of the prior.

#### Estimation of emergence rates from exit-trap data

Unfed females caught in exit-traps are assumed to be newly emerged nulliparous mosquitoes since *ad hoc* dissections of unfed exiting and resting females indicated that, as was the case in Muheza (Gillies & Wilkes, 1965) the great majority are newly emerged virgin females. We therefore assume that this was also the case among non-dissected insects so an estimate of the emergence rate on night *t*, $\lambda _t^{\left( {\rm E} \right)}$, is provided directly by the number of unfed mosquitoes caught in the exit-trap on the same night. $u_t^{\left( E \right)}$, the number of mosquitoes starting the gonotrophic cycle each night is then the sum of these emerging mosquitoes and survivors from the previous gonotrophic cycle. In the absence of delay between oviposition and host seeking ( ${a_0} = 1$) this is:

(11) $$\matrix{ {u_t^{\left( E \right)} = \lambda _t^{\left( E \right)} + g_t^{\left( E \right)}/{\Upsilon ^{\left( 1 \right)}}.} \cr }$$

where ${\rm g}_t^{\left( E \right)}$ is the expected number of gravid mosquitoes in an exit-trap, ${\Upsilon ^{\left( 1 \right)}}$ is a scale-factor equal to the trapping efficiency of gravids relative to emerging mosquitoes, with the unfed mosquitoes on the same scale as the emerging ones. In the general case where $0 < {a_0} < 1$, some host-seeking mosquitoes completed their previous cycle on the previous night and the estimate of the number of unfed mosquitoes (scaled by the unidentifiable trapping efficiency for gravid mosquitoes) is therefore:

(12) $$\matrix{ {u_t^{\left( E \right)} = \lambda _t^{\left( E \right)} + \; \left( {{a_0}g_t^{\left( E \right)} + \left( {1 - {a_0}} \right)g_{t - 1}^{\left( E \right)}} \right)/{\Upsilon ^{\left( 1 \right)}}} \cr }$$

#### Estimation of the duration of the resting period from exit-trap data

Following equivalent notation to Eq. (9) and assuming the resting period to correspond to a normal kernel (of mean $\theta _r^{\left( 4 \right)}$ and variance $\sigma _r^2$), the expected number of gravid mosquitoes in an exit-trap is a lagged function of the number of unfed mosquitoes at the start of the resting period so that:

(13) $$\matrix{ {g_t^{\left( E \right)} = {P_r}\; {\Upsilon ^{\left( 1 \right)}}\mathop \sum \limits_{\tau = 1}^{\tau = 4} \Phi _\tau ^{\left( E \right)}u_{t - \tau }^{\left( E \right)},} \cr }$$

where ${P_r}$ is the probability of surviving the resting period, $\tau$ is the lag in days and $\Phi _\tau ^{\left( E \right)}$ is the slice of the normal kernel assigned to $\tau$, so that $\Phi _\tau ^{\left( E \right)} = \Pr \left( {\tau - 0.5 < \theta _r^{\left( 4 \right)} < \; \tau + 0.5} \right)$. Substituting this into Eq. (12) and rearranging gives:

(14) $$\matrix{ {{g_t} = {{\rm P}_{\rm r}}\left( {{\Upsilon ^{\left( 1 \right)}}\mathop \sum \limits_{\tau = 1}^{\tau = 4} \Phi _\tau ^{\left( E \right)}\lambda _{t - \tau }^{\left( {\rm E} \right)} + {a_0}\mathop \sum \limits_{\tau = 1}^{\tau = 4} \Phi _\tau ^{\left( E \right)}{g_{t - \tau }} + \left( {1 - {a_0}} \right)\mathop \sum \limits_{\tau = 1}^{\tau = 4} \Phi _\tau ^{\left( E \right)}{g_{t - \tau - 1}}} \right).} \cr }$$

For the estimation of the duration of the resting period the total number of gravid females in exit-traps on night *t* was treated as a negative binomially distributed random variable with dispersion parameter ${r^{\left( 2 \right)}}$:

(15) $$\matrix{ {G_t^{\left( E \right)}\sim {\rm negbin}\left( {E\left( {G_t^{\left( E \right)}} \right),{r^{\left( 2 \right)}}} \right).} \cr }$$

where the expected number, $E\left( {{\rm G}_t^{\left( E \right)}} \right)$, is the product of the number of exit-traps, $n_t^{\left( E \right)},$ and the per-trap expectation, $E\left( {{\rm G}_t^{\left( E \right)}} \right) = n_t^{\left( E \right)}{g_t},$ and the empirical means were substituted for the lagged expected numbers of gravids, *i.e*.,:

(16) $$\matrix{ {{\rm g}_{t - \tau }^{\left( E \right)} = \displaystyle{{G_{t - \tau }^{\left( {\rm E} \right)}} \over {n_{t - \tau }^{\left( {\rm E} \right)}}}.} \cr }$$

An MCMC algorithm in rjags was used to obtain ${\Upsilon ^{\left( 1 \right)}}$, $\theta _r^{\left( 4 \right)}$ and the estimate, $\sigma _r^{\left( E \right)}$, of ${\sigma _r}$ with a uniform (1,5) prior for $\theta _r^{\left( 4 \right)}$, and gamma priors for $\displaystyle{1 \over {\sigma _r^{\left( E \right)2}}}$, ${\Upsilon ^{\left( 1 \right)}}$ and for the dispersion parameter ${r^{\left( 2 \right)}}$.

The fixed value of ${a_0}$ from the analysis of light-trap catches was used to compute $\theta _r^{\left( 4 \right)}$ by substituting $\theta _r^{\left( 4 \right)}$ into Eq. (2). Separate analyses were conducted in which either (i) an estimate, $P_r^{\left( 2 \right)}$, of ${P_r}$ was obtained by setting it equal to $\; P_o^{\left( 2 \right)}$, the estimate of survival per cycle from the analysis of light-trap data (*i.e*., neglecting mortality while ovipositing or host-seeking), or (ii) A value $P_r^{\left( 3 \right)}$ for ${P_r}$ was independently estimated from the exit-trap data, using a uniform (0,1) prior. Corresponding to the latter analysis, the estimates $P_r^{\left( 3 \right)}$ and $\theta _o^{\left( 4 \right)}\;$ were substituted into Eq. (3) to give an estimate ${p^{\left( 4 \right)}}$ of $p$.

#### Estimation of survival of male mosquitoes

The daily survival of male mosquitoes, ${\rm \pi }$, was obtained based on the emergence rate estimates (Eq. (11) or directly from the numbers of unfed mosquitoes in the exit-traps (see above)), based on the assumptions that emergence of male mosquitoes is proportional to that of female mosquitoes, and that both survival and sampling of male mosquitoes are age independent. The male mosquitoes emerging on each night define separate age-cohorts, with the average number of males per trap, ${v_t}$, representing the sum of the survivors for each cohort, so that:

(17) $$\matrix{ {{v_t} = \mathop \sum \limits_{\tau = 0}^{\tau = \infty } {{\rm \pi }^\tau }\lambda _{{\rm t} - \tau }^{\left( E \right)}.} \cr }$$

The terms in the summation become negligible for high $\tau$, so an upper bound to $\tau$ can be adopted, for which reasonable values depend on ${\rm \pi }$. The total number of males captured in exit-traps $V_t^{\left( {\rm E} \right)}$ can then be treated as a negative binomial variate:

(18) $$\matrix{ {V_t^{\left( {\rm E} \right)}\sim {\rm negbin}\left( {{{\Upsilon }^{\left( 2 \right)}}n_t^{\left( E \right)}v_t^{\left( E \right)},{r^{\left( 3 \right)}}} \right),} \cr }$$

where ${{\Upsilon }^{\left( 2 \right)}}$ is the trapping efficiency of males relative to nulliparous females; $n_t^{\left( E \right)}$ is the number of exit-traps; and ${r^{\left( 3 \right)}}$ is a further negative binomial dispersion parameter.

#### Simulation experiments to evaluate estimation of ${\theta _r}$ and ${P_r}$ from time-series analysis of parous rates

A set of 200 datasets were simulated, each the same size as the field datasets on parous rates, and with equivalent patterns of missing data, but with parameter values randomly sampled from distributions chosen to cover the entire plausible range for each parameter. Thus, parameters ${\theta _r},$ ${r^{\left( 1 \right)}}$, and ${P_r}\;$ were independently sampled from uniform (1.9,4.1), uniform (1,5), and uniform (0,1) distributions respectively and $1/{\sigma ^2}$ was sampled from a uniform (0.5,1) distribution. For each simulated dataset, values of ${\mu _t}$ were generated by sampling from a log-normal distribution with mean and variance matched to that estimated from the field data and the vector of values of for $T_t^{\left( L \right)}$ was obtained from Eq. (9). Simulated numbers of parous mosquitoes were then generated by inverting each of Eqs. (6)–(8).

The performance of the fitting algorithm in recovering the input parameters was evaluated using the same rjags functions as the primary analyses.

#### Simulation experiments to evaluate estimation of ${\theta _r}$ and ${P_r}$ from exit-trap data

A total of 50 datasets were simulated, each comprising data for a total of 207 days with complete data, with equivalent patterns of missing data to those in the first 3 years of exit-trap collections, and with parameter values randomly sampled from distributions chosen to cover the entire plausible range for each parameter. In each case, estimations were carried out for five different implementations of the models of Eqs. (11)–(18), varying in which parameters were treated as known, and which were estimated by the MCMC algorithm.

A further set of 100 simulated datasets were created in which, the number of days with complete data was varied by sampling the patterns of missing data from different subsets of the overall dataset. These datasets were used to investigate the effect of sample size on the parameter estimates.

## Results

### Data description

The overall sampling period spanned 8 years from January 2001 until January 2009, with the trapping effort and the numbers of *An. funestus* caught by each method given in Table 2. All of the *An. funestus* group mosquitoes examined morphologically had a single pale spot on the upper branch of the 5th vein and did not have a pale spot at the tip of the 6th vein and hence corresponded to *An. funestus*. All 71 females of the *An. funestus* group identified by PCR using the protocols of Koekemoer et al. (2002) were *An. funestus* (Charlwood, 2017). Since this is the most endophilic member of the species group, and that it was this behavior that was examined, we assume that this was the only member of the species group present in our collections.

**Table 2:** Trapping effort and numbers of *An. funestus* caught. Trap-nights refers to the total number of traps placed over all distinct nights.

	Exit	Light	Resting
Trapping effort			
Locations	421	881	222
Distinct nights	1,331	1,475	400
Trap-nights	7,396	6,041	1,527
Numbers of mosquitoes captured (Williams’ mean per trap night)^1^			
Unfed	72,838 (5.6)	290,561 (22.0)	3,769 (1.5)
Part fed	601 (0.04)	648 (0.05)	935 (0.29)
Fed	713 (0.04)	5,790 (0.31)	6,379 (1.6)
Semi gravid	936 (0.05)	212 (0.02)	791 (0.20)
Gravid	86,378 (5.5)	4,489 (0.27)	5,121 (1.9)
Male	496,445 (41.7)	11,056 (0.52)	13,190 (4.8)

**Note:**

1 The Williams’ mean of *x*, computed as ${\rm exp}\displaystyle{{\left( {\sum \ln \left( {x + 1} \right)} \right)} \over n} - 1,$ (where $n$ is the number of observed trap-nights) Is a measure of central value that can incorporate counts of 0 and has low sensitivity to extreme outliers.

Of the 71 unfed females dissected from resting collections 68 were virgins (Charlwood, Thompson & Madsen, 2003).

**Figure 1:**
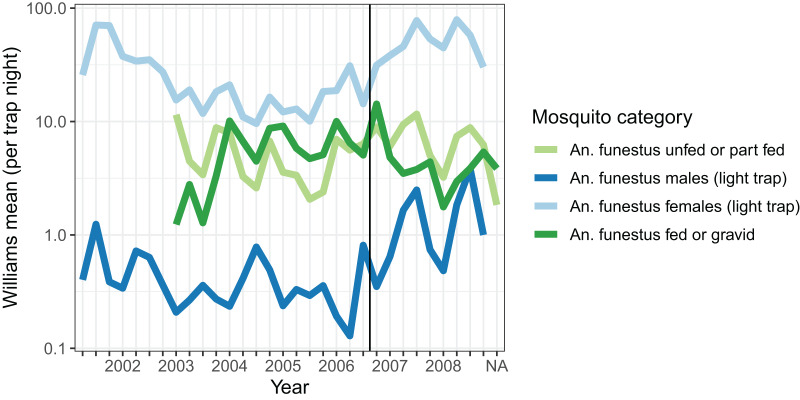
Average mosquito catch. The vertical line corresponds to the introduction of a cordon sanitaire of mosquito nets around the breeding site.

Densities of host-seeking mosquitoes were measured using light-trap collections throughout the period, and were highest in 2001, and then again in 2007 and 2008 (Fig. 1). Dissections were carried out on mosquitoes caught in light-traps between May 2007 and October 2008 on 87 distinct nights. Of 3,192 parous mosquitoes dissected, 1,927 (60.4%) had evident ovariolar sacs.

Resting catches were carried out intermittently throughout the research period, while exit-trap collections began in March 2003. Resting collections comprised mainly fed, gravid, or male mosquitoes, while many more unfed than fed mosquitoes were caught in exit-traps (Table 2). The majority of mosquitoes caught were in exit-traps and most of these were males. All female mosquitoes caught in exit-traps were analysed for blood meal status. The numbers of gravid females captured in exit-traps were comparable to those unfed or part-fed (Fig. 1).

**Figure 2:**
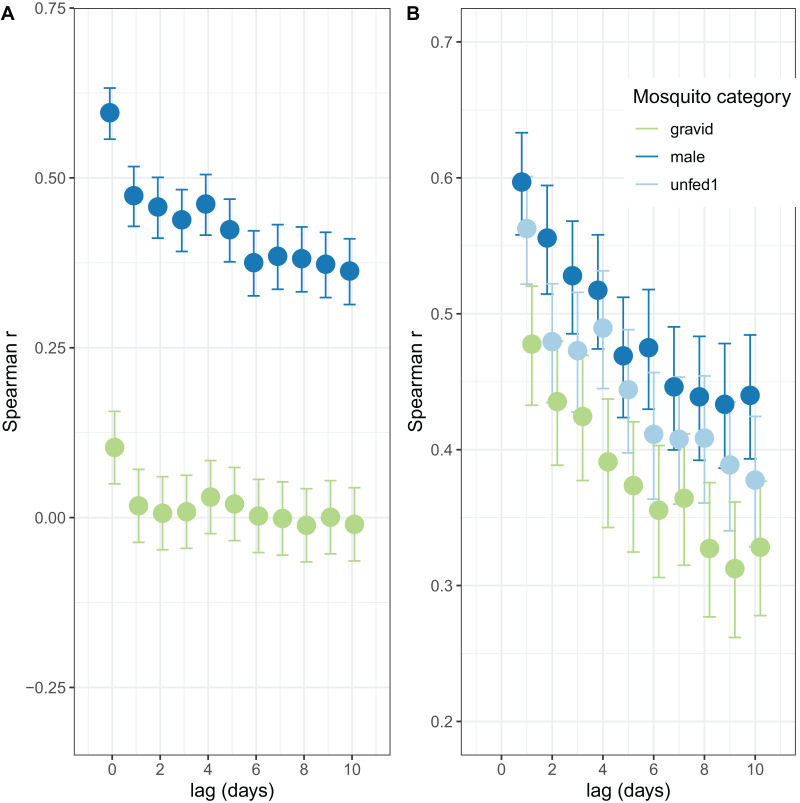
Correlation structure of exit-trap data. (A) Lagged correlations of mean (per night) exit-trap catches of male and of gravid mosquitoes with host-seeking (unfed or part fed) mosquitoes. (B) Lagged auto-correlations of mean numbers of mosquitoes caught in exit-traps. Points for the different categories are jigged to avoid overprinting.

The autocorrelations show the expected pattern of a decrease in correlation the longer the lag period (Fig. 2). The number of male mosquitoes in exit-traps was highly correlated with the number of mosquitoes that were still host-seeking (unfed or part-fed) on the same night. Lagged correlations between numbers of males and unfed/part-fed mosquitoes decreased with the time-interval (Fig. 2A). In contrast, there was only a small correlation between numbers of gravid mosquitoes in exit-traps and the numbers of unfed mosquitoes on the same night, and the lagged correlations were close to zero (Fig. 2A). All three categories of mosquitoes showed a strong temporal autocorrelation in mean numbers in exit-traps (Fig. 2B). This was highest for males (consistent with the same age-cohorts of male mosquitoes being represented on successive nights), lowest for gravid mosquitoes (each age cohort is gravid only once per cycle), and intermediate for unfed/part-fed mosquitoes, for which the autocorrelation is likely to reflect temporal correlations in environmental factors.

Additional, complementary, descriptions of the temporal patterns of mosquito densities and numbers of mosquitoes in different categories over time are provided by Charlwood (2017).

### Evaluation of estimators using simulations

Simulated datasets were generated with the same numbers of traps sampled on each day as in the corresponding field data, and with autocorrelation structure based on that found in the data. These datasets were used to evaluate the performance of the different estimators.

**Figure 3:**
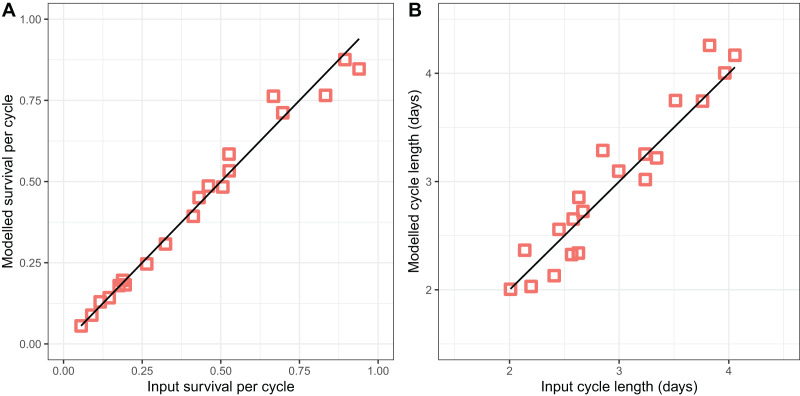
*Performance of model for light-trap data in simulations*. (A) Comparison of estimated survival per cycle ${\rm P_O}^2$ with input value. (B) Comparison of estimated cycle length $\theta_{\rm O}^3$ with input value [i].

The modified version of the Birley model (Eqs. (2)–(5)) fitted to the light-trap data (Fig. 3) performed well in recovering the parameter values used to generate the simulated datasets, accurately recovering both the survival per cycle (Fig. 3A) and the duration of the oviposition cycle (Fig. 3B), over the entire range of values simulated. The estimates of both variables appear to be unbiased, but the estimates of cycle duration displayed more of a tendency to deviate from the target (input) values.

The performance of the novel model fitted to exit-trap data for female mosquitoes (Eqs. (11)–(16)) depended on which of the parameters were assumed known from other data (*e.g*., from concurrent light-traps) with five different implementations evaluated (a–e in Table 3). These five implementations differed in which parameters were estimated, and which were assumed known from other data (Table 3).

**Table 3:** Female *An. funestus*: evaluation of estimates made from simulated exit-trap datasets.

			Implementation				
			a	b	c	d	e
Parameters fixed at true values			${P_r},{\rm \; }{a_0}$	${a_0}$	${a_0},{\Upsilon ^{\left( 1 \right)}}$	${a_0}$	${P_r}$
Parameters fixed at arbitrary values			–			${P_r} = 0.75$	${a_0}{\rm \; \; } = 0.3$
${\Upsilon ^{\left( 1 \right)}}$	bias		0.236 (0.036, 0.437)	0.093 (−0.009, 0.196)	0*	−0.241 (−0.413, −0.069)	0.238 (0.038, 0.438)
${\Upsilon ^{\left( 1 \right)}}$	CCC		0.402 (0.259, 0.528)	0.497 (0.339, 0.628)	1*	0.156 (0.031, 0.276)	0.405 (0.262, 0.530)
$P_{\rm r}^{\left( 3 \right)}$	bias		0*	1.880 (−0.418, 4.180)	0.169 (−0.014, 0.351)	3.170 (1.010, 5.330)	0*
$P_{\rm r}^{\left( 3 \right)}$	CCC		1*	0.843 (0.776, 0.891)	0.993 (0.990, 0.995)	–	1*
$\theta _r^{\left( 4 \right)}$	bias		0.055 (−0.001, 0.111)	0.067 (0.007, 0.126)	0.050 (−0.006, 0.105)	−0.062 (−0.125, 0.001)	0.047 (−0.007, 0.101)
$\theta _r^{\left( 4 \right)}$	CCC		0.800 (0.720, 0.859)	0.787 (0.704, 0.849)	0.801 (0.721, 0.860)	0.764 (0.671, 0.833)	0.804 (0.727, 0.861)

**Note:**

* Estimate necessarily corresponds to input value; CCC is concordance correlation coefficient.

By assuming externally determined values of female mosquito survival, ( ${P_r}$) and of the sac rate, ${a_0}$, implementation a corresponds to the external data available in Furvela. Implementation b makes use only of an external value for the sac rate, ${a_0}$. Since it is reasonable to treat ${a_0}$ as invariant, this provides a way of estimating ${P_r}$ for subsets of the full dataset, for instance in order to evaluate annual variations. Implementation c, in which both ${a_0}$ and the trapping efficiency, ${\Upsilon ^{\left( 1 \right)}}$, are assumed known, removes one important source of uncertainty from the other implementations, while implementations d and e evaluate whether misspecification of the values of $P$ and ${a_0}$ respectively, have important effects on the estimates of the other parameters.

**Figure 4:**
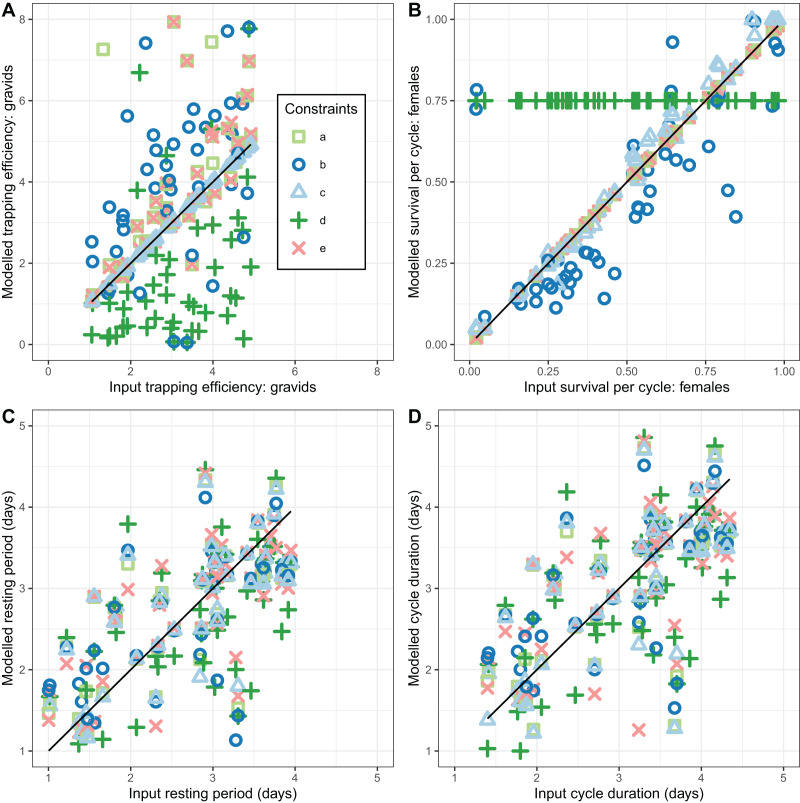
Performance of estimators for female mosquitoes. (A) Comparison of estimated trapping efficiency for gravid mosquitoes $\Upsilon^{(1)}$ with input value; (B) comparison of estimated survival of female *An. funestus* mosquitoes ${\rm P_r^{(2)}}$ with input value; (C) comparison of estimated duration of resting period $\theta_{\rm r}^{(4)}$ with input value; (D) comparison of estimated duration of full oviposition cycle $\theta_{\rm O}^{(4)}$ with input value. Results are shown for a total of 50 simulated datasets. The different implementations a–e are as described in Table 3.

None of the implementations gave estimates of ${\Upsilon ^{\left( 1 \right)}}$ that reliably agreed with the simulated values (CCC values were all less than 0.5 corresponding to considerable scatter in Fig. 4A). Implementations a, b, and e gave estimates with a positive bias while implementation d gave estimates with negative bias (Table 3). Precise and unbiased estimates of female mosquito survival were only obtained by fixing ${\Upsilon ^{\left( 1 \right)}}\;$ at its true value (implementation c) (Fig. 4B). When only ${a_0}$ was fixed at the true value (implementation b) $P_{\rm r}^{\left( 2 \right)}$ was often a poor estimate, with the bias (mean 1.9) having wide confidence intervals, although these overlapped with zero. The overall level of agreement between estimates and true values was nevertheless acceptable (CCC = 0.84) (Table 3). All five of the implementations deliver comparable performance in estimating cycle length ( $\theta _o^{\left( 4 \right)}$). These estimates are close to unbiased (with narrow confidence intervals overlapping 0 for the bias), and with an acceptable CCC of around 0.8, though for many of the individual simulations the estimates were quite poor (Fig. 4).

**Figure 5:**
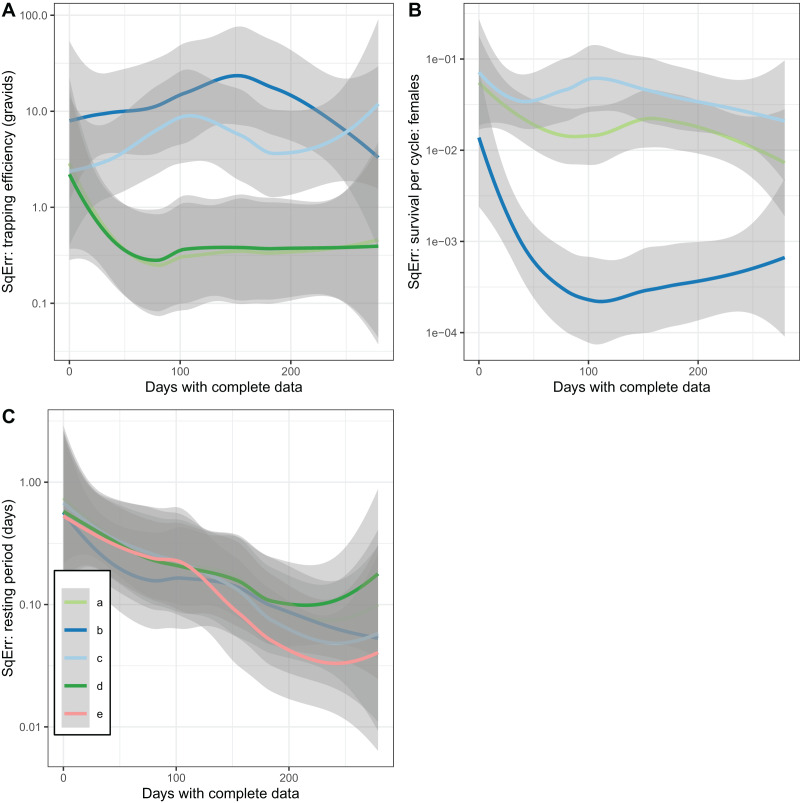
Consistency of estimates for female mosquitoes. Loess-smoothed estimates of squared differences between values input to simulations and estimated values as functions of the size of the dataset. Shading indicates 95% confidence regions; (A) trapping efficiency for gravid mosquitoes; (B) survival of female mosquitoes per cycle; (C) duration of resting period. Implementation a: survival and sac rate both known; b: sac rate known, survival estimated; c: trapping efficiency and sac rate known, survival estimated.

In addition to accuracy and a lack of bias, a further desirable property of an estimator is that it converges with the true value as the sample size is increased (consistency). This was not achieved by the estimates of trapping efficiency, for which there was no decrease in error as the size of the simulated dataset increased (Fig. 5A). The error did show the expected decrease with increased sample size in the estimates of survival per cycle. The error is already very small with 60 or more days with complete data, when the trapping efficiency is known. When trapping efficiency is estimated a very large number of nights of collection appear to be needed to ensure accuracy of the estimates (Fig. 5B). Although the methods do not require all the collection nights to be consecutive, a need for 2 months or more of data is likely to be a severe limitation. Irrespective of which of the other parameters were treated as known, the error in estimates of the duration of the resting period or of the full cycle decreased with an increase in sample size up to a value of about 100 days with complete data. Above this sample size there was no further improvement (Fig. 5C).

**Figure 6:**
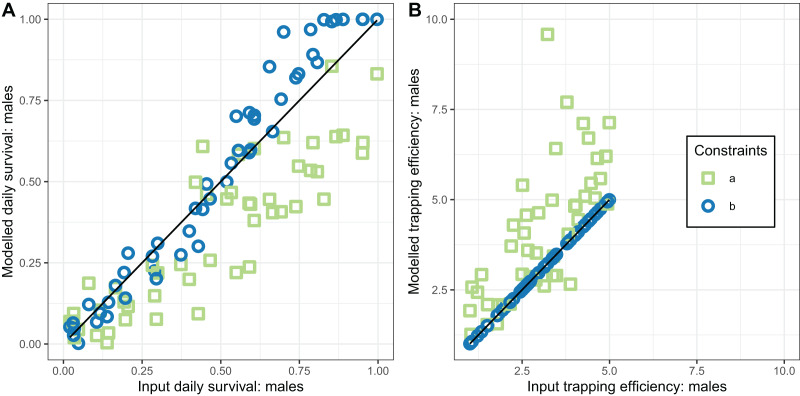
Performance of estimators for male mosquitoes. (A) Comparison of estimated survival of male mosquitoes with input value; (B) comparison of estimated trapping efficiency for male mosquitoes with input value. a and b indicate the implementation as described in Table 4.

The performance of the novel model fitted to exit-trap data for male mosquitoes (Eqs. (17) and (18)) was evaluated for two implementations, differing in whether the trapping efficiency for males, ${\Upsilon ^{\left( 2 \right)}}$, was assumed to be known. When ${\Upsilon ^{\left( 2 \right)}}$ was estimated (implementation a) these estimates were very inaccurate and hardly correlated with the true value (Table 4 and Fig. 6).

**Table 4:** Male mosquitoes: evaluation of estimates made from simulated datasets.

		Implementation	
		a: trapping efficiency estimated	b: trapping efficiency ${\Upsilon ^{\left( 2 \right)}}$ fixed at true value
${\Upsilon ^{\left( 2 \right)}}$	bias	1.57 (−0.061, 3.20)	0*
${\Upsilon ^{\left( 2 \right)}}$	CCC	0.004 (−0.039, 0.046)	1*
$\pi$	bias	−0.155 (−0.328, 0.019)	0.068 (−0.031, 0.167)
$\pi$	CCC	0.767 (0.653, 0.847)	0.961 (0.940, 0.976)

**Note:**

* Estimate necessarily corresponds to input value.

In contrast, the estimates of male survival were reasonably accurate even when ${\Upsilon ^{\left( 2 \right)}}$ was unknown. They were very highly accurate when ${\Upsilon ^{\left( 2 \right)}}$ was known.

**Figure 7:**
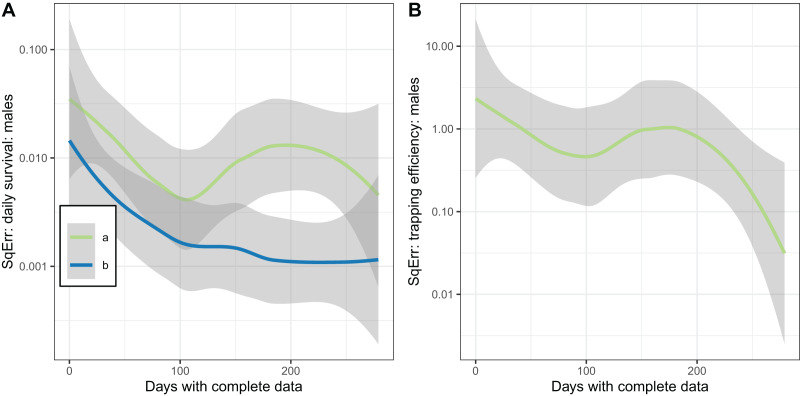
Consistency of estimates for male mosquitoes. Loess-smoothed estimates of squared differences between values input to simulations and estimated values as functions of the size of the dataset. Shading indicates 95% confidence regions; (A) daily survival of male mosquitoes; (B) trapping efficiency of male mosquitoes. a and b indicate the implementation as described in Table 4.

The plots of the squared error of the estimates against the sample size (Fig. 7) indicated the expected improvement with increased sample size for the implementation with fixed ${\Upsilon ^{\left( 2 \right)}}$. The relationship was less clear when ${\Upsilon ^{\left( 2 \right)}}$ was estimated.

### Parameter estimation from aggregated data only

The estimates of life cycle parameters from aggregated data (Table 5) had narrow confidence intervals, reflecting the large number of observations. The estimated average duration of the resting period was close to 2 days, with slightly higher values obtained when resting period mortality was allowed for. Less than half of the female mosquitoes were estimated to survive the full cycle.

**Table 5:** Estimates of life cycle parameters from aggregated data.

Symbol	Description	Units	Method (reference)	Estimate	Value (95% CL)
${{\rm P}_{\rm o}}$	Survival of adult female mosquitoes per oviposition cycle	Proportion	Davidson (1954)	$P_o^{\left( 1 \right)}$	0.481 [0.469–0.493]
${\theta _r}$	Mean duration of resting period	Days	Kulkarni et al. (2006), Tchuinkam et al. (2010)	$\theta _r^{\left( 1 \right)}$	1.93 [1.89–1.96]
			Kulkarni et al. (2006), Tchuinkam et al. (2010)^1^	$\theta _r^{\left( 2 \right)}$	2.23 [2.18–2.28]
${a_0}$	Proportion of host-seeking mosquitoes that return on the same night as oviposition	Proportion	Charlwood et al. (1985)	${a_0}$	0.604 [0.586–0.621]
${\theta _o}$	Mean duration of full oviposition cycle	Days	Kulkarni et al. (2006), Tchuinkam et al. (2010)	$\theta _o^{\left( 1 \right)}$	2.58 [2.53–2.64]
			Kulkarni et al. (2006), Tchuinkam et al. (2010)^1^	$\theta _o^{\left( 2 \right)}$	2.88 [2.82–2.95]
$p$	Daily survival of female mosquitoes	Proportion	Eq. (3)	${p^{\left( 1 \right)}}$	0.753 [0.745–0.761]
			Eq. (5) ^1^	${p^{\left( 2 \right)}}$	0.776 [0.768–0.783]

**Note:**

1 With adjustment for resting stage mortality.

### Parameter estimation from time-series data

Fitting of the model of Eqs. (6)–(9) to the field data from light-traps gave an estimate of $P_{\rm o}^{\left( 2 \right)}$ = 0.528, which is rather higher than the unadjusted parous rate of $P_{\rm o}^{\left( 1 \right)}$ = 0.481 (Table 6).

**Table 6:** Point and interval estimates of quantities derived time-series analysis of light-trap data.

	Meaning	Eqn.	Estimate (95% CI)
$P_{\rm o}^{\left( 2 \right)}$	Proportion of female mosquitoes surviving the oviposition cycle	(9)	0.528 [0.461–0.612]^1^
$\theta _o^{\left( 3 \right)}$	Oviposition cycle duration (days) from light-traps	(9)	2.13 [1.55–2.85]^1^
$\theta _r^{\left( 3 \right)}$	Resting period duration (days) from light-traps	(2), (9)	1.47 [0.80–2.15]^3^
${p^{\left( 3 \right)}}$	Proportion of female mosquitoes surviving each day	(1)	0.740 [0.650–0.815]^2^
${{r}^{\left( 1 \right)}}$	Overdispersion parameter for the negative binomial distribution of numbers of parous mosquitoes.	(3)	0.785 [0.137–3.004]^1^
$\sigma _o^{\left( 1 \right)}$	Standard deviation of the duration of the oviposition cycle (days)	(5)	0.508 [0.354–0.770]^1^

**Notes:**

1 Estimated by fitting to the entire light-trap dataset by MCMC.

2 Sample-based confidence intervals.

3 Using the sac rate (Eq. (2)) to estimate the difference between $\theta _r^{\left( 3 \right)}$ and $\theta _o^{\left( 3 \right)}$.

$P_{\rm o}^2$ = 0.528 is to be preferred to the crude parous rate as an estimate of survival per cycle, because it does not depend on the approximation that an integral number of seasonal cycles were representatively sampled. The small difference between the crude and model-based survival estimates is consistent with a modest level of unrepresentativeness in the sampling. The same model also gave an estimate of only 2.13 days for the oviposition cycle duration (Table 6) which is shorter than the estimate based on the resting collection data only. The corresponding estimate of daily survival of *p* = 0.74, corresponding to life expectancy of the adult female mosquito assuming an exponential survival model of ln(2)/ln(*p*) = 2.3 days (95% credible intervals [1.6–3.4]). The numbers of parous mosquitoes show only a limited degree of overdispersion and the estimate of the standard deviation of the duration of the oviposition cycle based on light-trap data, $\sigma_o^{(1)}$, is also low (Table 6).

The numbers of female mosquitoes captured in exit-traps were analysed with three different implementations of the model of Eqs. (10)–(15) (Table 7). Implementation a, which used the estimate of survival from the analysis of light-trap data, gave an estimate of ${{\Upsilon }^{\left( 1 \right)}} = 1.8$, implying that a gravid mosquito is almost twice as likely to be caught in exit-traps as an emergent female. In contrast, when the model was used to estimate $P$, very high survival estimates were obtained but the trapping efficiency was estimated to be about 0.4. In view of the evidence from simulations that fixing $P$ leads to overestimation of ${{\Upsilon }^{\left( 1 \right)}}$, and of the absence of other evidence for a difference in trapping efficiency between emergent and gravid females, a third set of estimates were made, fixing ${{\Upsilon }^{\left( 1 \right)}}$ at a value of 1. These gave intermediate levels of survival and similar estimates of the duration of the oviposition cycle to those obtained from the light-trap data.

**Table 7:** Parameter estimates for female *An. funestus* mosquitoes from fits to the complete exit-trap dataset.

		Implementation		
Symbol	Meaning	a	b	c
$P_{\rm o}^{\left( 3 \right)}$	Proportion of female mosquitoes surviving the oviposition cycle	0.528*	0.890 (0.746, 0.992)	0.704 (0.648, 0.766)
${p^{\left( 4 \right)}}$	Proportion of female mosquitoes surviving each day	0.749 (0.589, 0.813)	0.941 (0.834, 0.996)	0.840 (0.711, 0.895)
$\theta _r^{\left( 4 \right)}$	Duration of the resting period in days	1.810 (0.810, 2.690)	1.538 (0.655, 2.354)	1.626 (0.671, 2.500)
$\theta _o^{\left( 4 \right)}$	Duration of the oviposition cycle in days	2.206 (1.207, 3.086)	1.934 (1.051, 2.750)	2.023 (1.068, 2.896)
$\sigma _r^{\left( 1 \right)}$	Standard deviation of duration of the resting period	2.00 (1.11, 4.56)	2.21 (1.21, 4.88)	2.06 (1.11, 4.71)
${{\Upsilon }^{\left( 1 \right)}}$	Trapping efficiency of gravid, relative to emergent females	1.784 (1.471, 2.124)	0.403 (0.183, 0.786)	1*

**Note:**

* Pre-assigned parameter value.

The numbers of male mosquitoes captured in exit-traps were analysed with two different implementations of the model of Eqs. (16) and (17) (Table 8), with implementation b constraining the trapping efficiency for male mosquitoes to be the same as that for emergent females.

**Table 8:** Parameter estimates for male mosquitoes from fits to the complete exit-trap dataset.

		Implementation	
Symbol	Meaning	a	b
${\rm \pi }$	Daily survival of male mosquitoes	0.649 (0.565, 0.739)	0.939 (0.863, 0.995)
${{\Upsilon }^{\left( 2 \right)}}$	Trapping efficiency of males relative to emergent females	2.908 (2.404, 3.462)	1*

**Note:**

* Pre-assigned parameter value.

**Figure 8:**
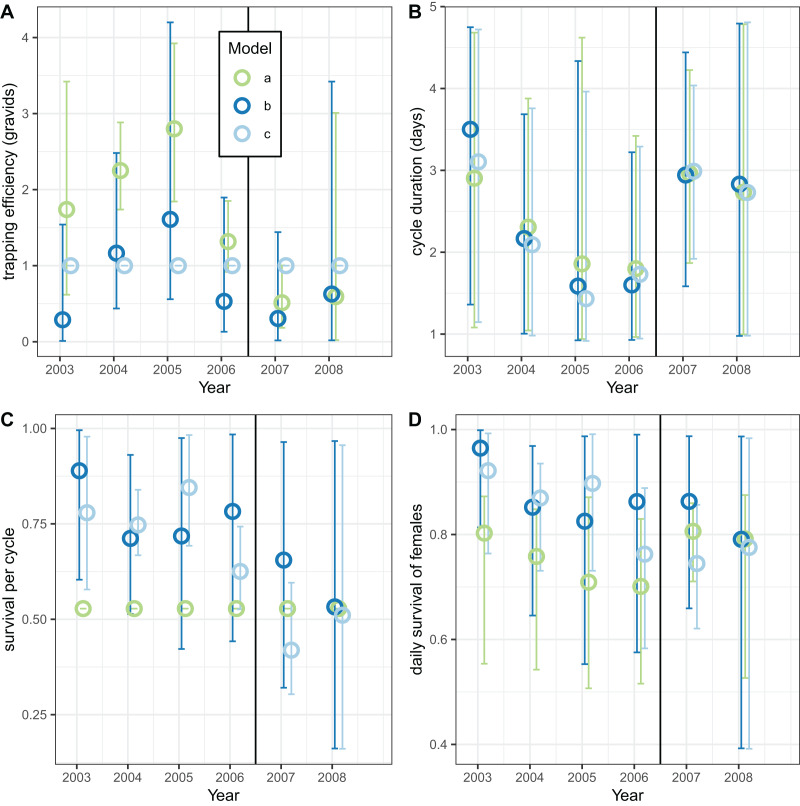
Estimates for female mosquitoes derived by fitting to exit-trap data separately for each year. (A–D) Correspond to different outcomes; colours correspond to different implementations: a: estimate of survival per cycle (P) taken from model for light-traps; b: trapping efficiency and survival both estimated; c: trapping efficiency = 1 (emerging and gravid females assumed equally likely to be caught). In each case the sac rate is taken from the analysis of light-trap data. Error bars are 95% credible intervals.

Analyses of the exit-trap data for female mosquitoes were made separately for each annual period (Fig. 8) using the same model implementations for the global analyses reported in Tables 7 and 8. When the trapping efficiency was not constrained, the estimates of ${{\Upsilon }^{\left( 1 \right)}}$ for the different years varied considerably, with a maximum in 2005 suggesting that the introduction of LLINs was associated with a reduction in ${{\Upsilon }^{\left( 1 \right)}}$ (Fig. 8A). Irrespective of assumptions about trapping efficiency or survival, the estimated of cycle duration decreased from 2003–2006 but then increased again (Fig. 8B) although these estimates all have wide credible intervals. Correspondingly, the estimated survival per cycle (Fig. 8C) was lower in the later years. When the trapping efficiency was fixed (implementation c) there was more of an indication of the expected decrease between the first and second halves of the study period in estimates both of survival per cycle and daily survival (Fig. 8D).

**Figure 9:**
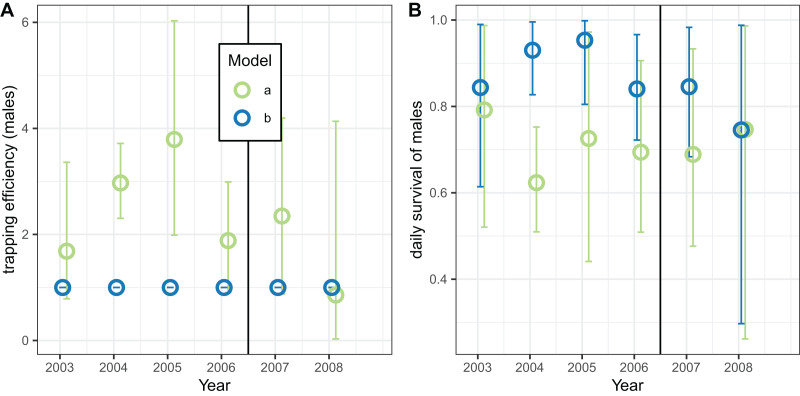
Estimates for male *An. funestus* mosquitoes derived by fitting to exit-trap data separately by year. (A and B) Correspond to different outcomes; colours to different implementations: a: trapping efficiency fixed at $\Upsilon = 1$; b: survival $\pi$ and $\Upsilon^{(2)}$ both estimated. Error bars are 95% credible intervals.

The corresponding analyses of data for male mosquitoes estimated uniformly very high daily survival ( ${\rm \pi }$) when ${{\Upsilon }^{\left( 2 \right)}} = 1$ (Fig. 9B). When ${{\Upsilon }^{\left( 2 \right)}}$ was allowed to vary, the estimates of this quantity were mostly substantially higher than unity, while the estimates of ${\rm \pi }$ were about 0.7 (a little higher than the global estimate of 0.65 (Table 8)), with no indication of any trend over time.

**Figure 10:**
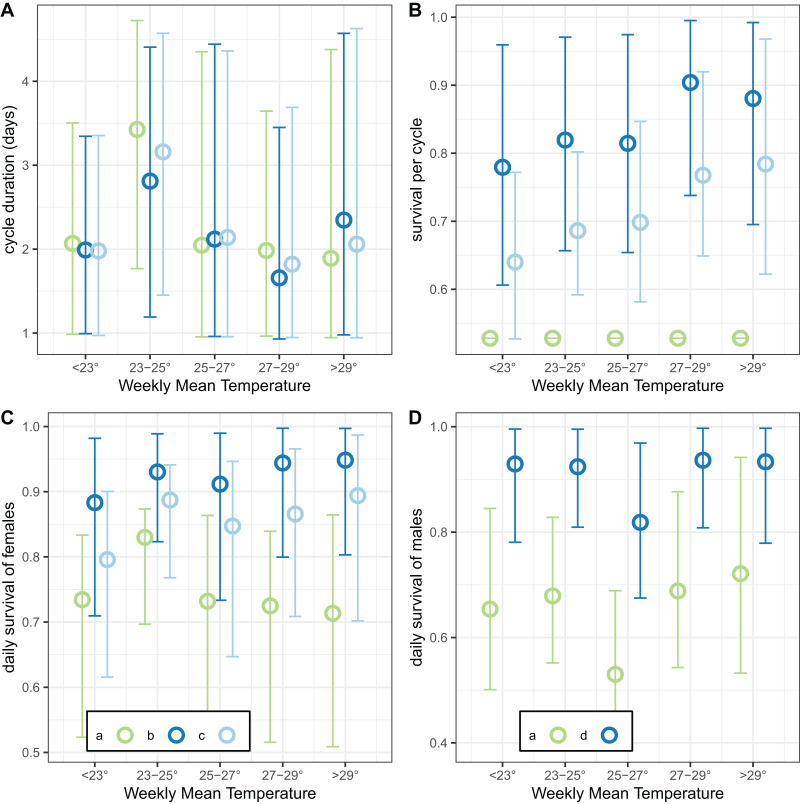
Temperature-dependent cycle length and survival estimates from exit-trap data. Model implementations: a: estimate of survival of females per cycle (P) taken from model for light-traps; b: trapping efficiency and survival of females both estimated; c: trapping efficiency for females = 1 (emerging and gravid females assumed equally likely to be caught); d: trapping efficiency for males = 1. In each case the sac rate is taken from the analysis of light-trap data. Error bars are 95% credible intervals.

The final analysis of the exit-trap data estimated separate values for cycle duration and survival by temperature (Fig. 10). The estimates of cycle duration were highest for weeks with mean temperatures of 23–25 °C, although all these estimates were very imprecise (Fig. 10A). The estimated female survival per cycle tended to increase with temperature but the confidence intervals were again broad (Fig. 10B).

Analysis of the linear trend on a logistic scale found that the odds of surviving the cycle were multiplied by 1.021 for every degree of mean weekly temperature increase (95% credible interval [0.991–1.051]). This modest trend was even less obvious when the results were expressed as survival per 24-h period (Fig. 10C). Male survival was almost independent of temperature (Fig. 10D), with the odds of surviving multiplied by only 1.002 per degree of mean weekly temperature increase (95% credible interval [0.967–1.040]).

## Discussion

Although there have been many innovations in sampling different components of malaria vector populations, estimation of life-cycle parameters for malaria vectors largely relies on a small repertoire of trapping technologies and statistical methods. Time-series analysis can provide estimates of quantities that are otherwise largely unavailable, such as the survival in nature of male mosquitoes, but is rarely applied. Moreover, time-series methods do not depend on representative sampling of mosquitoes over entire annual cycles, so they can be validly applied to data from relatively short time periods. This can be exploited for estimating impacts of temporally varying interventions, and for use in highly seasonal settings where sampling throughout the year is impracticable. The Furvela dataset is particularly rich because large numbers of houses were sampled using multiple methods over an extended period, with an innovative aspect being the deployment of large numbers of exit-traps. Exit-traps share with resting collections the advantage that they do not require electrical power, and so a large number of traps can be deployed over a wide area, irrespective of power availability, making it possible to analyse local variation.

Since mosquitoes are ectothermic, their bionomic parameters are expected to be temperature dependent, and shorter cycle durations at higher temperatures can be demonstrated in the insectary (Lardeux et al., 2008). For instance, an insectary study found an optimal temperature for adult survival of 25 °C (in *An. gambiae* s.s.) (Christiansen-Jucht et al., 2014) and temperature sensitivity of *An. funestus* in the laboratory is broadly similar to that of members of the *An. gambiae* complex (Lyons et al., 2012) but with a few exceptions (*e.g*., Gillies & Wilkes, 1963) field studies and transmission models have ignored this variation. Densities of *An. funestus* in Furvela increased strongly with temperature (Charlwood, 2017), but surprisingly, the temperature gradient in the cycle durations observed in insectaries is not seen in the exit-trap data. The modest increase in survival of females with temperature is also at variance with the laboratory findings. This might be because annual variations in temperature inside houses are much less than variations measured in weather stations (Paaijmans & Thomas, 2011) and could help explain the higher sporozoite rates during warmer periods (Charlwood, 2017). It will be interesting to see if this can be replicated elsewhere.

Male survival in nature is potentially an important quantity for modelling of population dynamics and of the impact of genetic control *via* population suppression, but most approaches for estimating female survival cannot be applied to males. In principle, mark-release-recapture methods might provide an alternative means of estimating survival (Epopa et al., 2017), but males are more fragile than females (as exemplified by their narrower thermal limits of survival (Lyons et al., 2012)) so conventional marking techniques are more likely to harm them. Analysis of the sex ratios observed in swarms (bearing in mind that females only mate once) might provide another way of estimating relative survival of males, but to the best of our knowledge, analyses of exit-trap data provide the only easily available estimates of male survival of Anopheles in nature. In contrast to female survival, it showed little variation either by temperature or year-to-year, consistent with the fact that males are not targeted by LLINs.

The analysis of simulation data revealed several challenges specific to this dataset with the statistical methods. Firstly, in Furvela the houses were not sampled representatively, and more representative sampling would be important if formal within-population comparisons are intended, such as the use of these methods for estimating intervention impacts in trials.

Secondly, the methods are sensitive to assumptions about the relative trapping efficiency for different stages of the mosquito life cycle. This introduces considerable uncertainty into our estimates of male survival, linked to the uncertainty in the trapping efficiency, ${{\Upsilon }^{\left( 2 \right)}}$. The results for females are broadly consistent with the hypothesis that emergent and gravid female insects are equally likely to be caught in an exit-trap. This makes sense with *An. funestus* since it is very highly endophilic and collecting inside houses should, theoretically, produce a uniform collection efficiency. *Anopheles gambiae* complex vectors are influenced by the weather—when it rains they enter houses as shown, (or at least implied) by paired tent/light collections in Ghana (Charlwood et al., 2011). This may be a reason why attempts to use the Birley model with *An. gambiae* have not produced the anticipated results. Unfed and gravid insects very likely use different openings to enter houses, so the construction of the house will also have a strong influence on within-house comparisons, especially because the domestic architecture in the area is highly variable. This justifies our approach of comparing numbers of different categories of mosquitoes across many houses rather than focusing on comparisons within houses. Where houses have mud or stone walls, as in Muheza, Tanzania, or the village of Massavasse 200 km to the south of Furvela, exit collections recovered many more semi-gravid mosquitoes than were collected in Furvela (Charlwood et al., 2013; Gillies, 1954). In Furvela intra-domestic movement of semi-gravid mosquitoes was observed but they did not exit. The temperature may also differ between different kinds of house.

Thirdly, established methods, including the original Birley approach (Birley & Rajagopalan, 1981) and the analysis of the fed:gravid ratio in resting collections, treat the resting period, ${\theta _r}$, (or the full cycle, ${\theta _o}$) as an integral constant. The time-series methods in this article treats the cycle duration as a distribution (as did Holmes & Birley (1987)), allowing for the likelihood that some mosquitoes take more nights to digest their blood meals than others. The high values estimated for the standard deviation of cycle duration, $\sigma _o^{\left( 1 \right)}$ and $\sigma _r^{\left( 1 \right)}$, support the notion that there is considerable variation in the duration of the cycle. The use of a normal kernel for this variation is broadly supported by published distributions for cycle duration (Beier, 1996) (although the duration of any individual cycle is best understood as an integral number of 24-h periods). Leaving to oviposit is a gated phenomenon so that a mosquito will only leave to oviposit at dusk. Thus, if she is gravid at midnight then she will have a considerable delay compared to a mosquito that is gravid at five o’clock in the afternoon. Experiments in Papua New Guinea found that the time of feeding did not affect cycle duration, at least in the *An. punctulatus* complex (Charlwood, Graves & Birley, 1986). Similarly, in Brazil there was no apparent difference in duration between *Nyssorhinchus darlingi* released fed and unfed (which implied that there was no real difficulty associated with obtaining a blood-meal) (Charlwood & Alecrim, 1989).

During each phase of the gonotrophic cycle mosquitoes experience different mortality risks. Vectorial capacity depends on the mosquito surviving the number of gonotrophic cycles needed to complete the intrinsic incubation period of the parasite. As pointed out by Hii (1985), Hii et al. (1985), Hii, Birley & Sang (1990) and Birley (1990), daily survival rates, determined by dissection, are remarkably similar between malaria vectors from different continents, which suggests that survival may be independent of the duration of the gonotrophic cycle. Cycle duration may, however, vary considerably with environmental factors. This may be one reason why our estimates of the mean duration of the gonotrophic cycle duration are relatively imprecise. However, survival estimates of malaria vectors also depend to a certain extent on the methods used (Matthews, Bethel & Osei, 2020). In their meta-analysis of a number of previous studies, Matthews, Bethel & Osei (2020) obtained survival rate from dissections (vertical) of 0.83 (95% CI [0.80–86]), similar to the results obtained during population declines in the absence of recruitment (Charlwood et al., 1985). MRR studies (that include survival and non-emigration) gave a value of 0.73 (95% CI [0.66–079]) and delayed infection rates (parasitological) gave 0.92, (CI [0.86–095]). Such differences translate into large differences in vectorial capacity. In the present analysis daily survival of females from exit-trap collection was 0.75 (CI [0.59–0.81]), which is considerably lower than that usually derived from parous rate determination. Nevertheless, malaria transmission in the village was intense. Transmission is also affected by the number of mosquitoes biting. Densities of *An. funestus* in Furvela were always considerable (Fig. 1) and it is probably this which accounted for the very high transmission given the relatively low survival rate of the mosquitoes.

In models that allow for it, variation between insects in cycle duration contributes additional uncertainty to estimates of mean cycle duration. It follows that estimates of this important parameter are probably not very reliable, irrespective of the estimation technique and this in turn lends uncertainty to the value for the daily survival of female mosquitoes, $p$, (which depends on ${\theta _o}$ *via* Eq. (3)) and hence to any estimates of the absolute value of the vectorial capacity that might be derived from these analyses. Studies that use survival of female mosquitoes as an outcome to be compared between geographical areas, or over time, should therefore consider inferring effects from relative survival of the different groups of mosquitoes, rather than calculating absolute values for cycle duration.

## Conclusions

Survival and gonotrophic cycle duration are important determinants of the vectorial capacity of malaria vectors but there are a limited number of approaches to estimate these quantities from field data. Analyses using existing and novel approaches to time-series data on *Anopheles funestus* mosquitoes caught over 7 years in Furvela, Mozambique, enabled the estimation of daily mosquito survival and the duration of the feeding cycle. The results suggest that male *An. funestus* have higher survival rates than females, and that male survival was temperature independent and unaffected by the introduction of long-lasting insecticidal nets. The patterns of temperature dependence in females are at variance with results of laboratory studies. Time series approaches have the key advantage for estimating survival that they do not depend on representative sampling of mosquitoes over the whole year. However, the estimates of oviposition cycle duration were associated with considerable uncertainty, which appears to be due to variability between insects in the duration of the resting period, and the estimates based on exit-trap data are sensitive to assumptions about relative trapping efficiencies.

## Supplemental Information

10.7717/peerj.15230/supp-1 Supplemental Information 1 Bias in parous rate estimates of survival per cycle.

10.7717/peerj.15230/supp-2 Supplemental Information 2 Parous rates in continuously growing or decreasing mosquito population.

## Additional Information and Declarations

### Data Availability

The following information was supplied regarding data availability: The data and code are available at GitHub and Zenodo: https://github.com/ThomasASmith/Furvela_Entomology, this repository includes a codebook that provides an English translation of Portuguese terminology. ThomasASmith. (2023). ThomasASmith/Furvela_Entomology: Entomology data and code for Furvela, Mozambique (v1.0). Zenodo. https://doi.org/10.5281/zenodo.7607159.
