# Supplementary figures and images for: Time series analysis of survival and oviposition cycle duration of Anopheles funestus (Giles) in Mozambique
Source: PeerJ. 2023 May 29;11:e15230. doi: 10.7717/peerj.15230 (PMC10234278; doi:10.7717/peerj.15230)

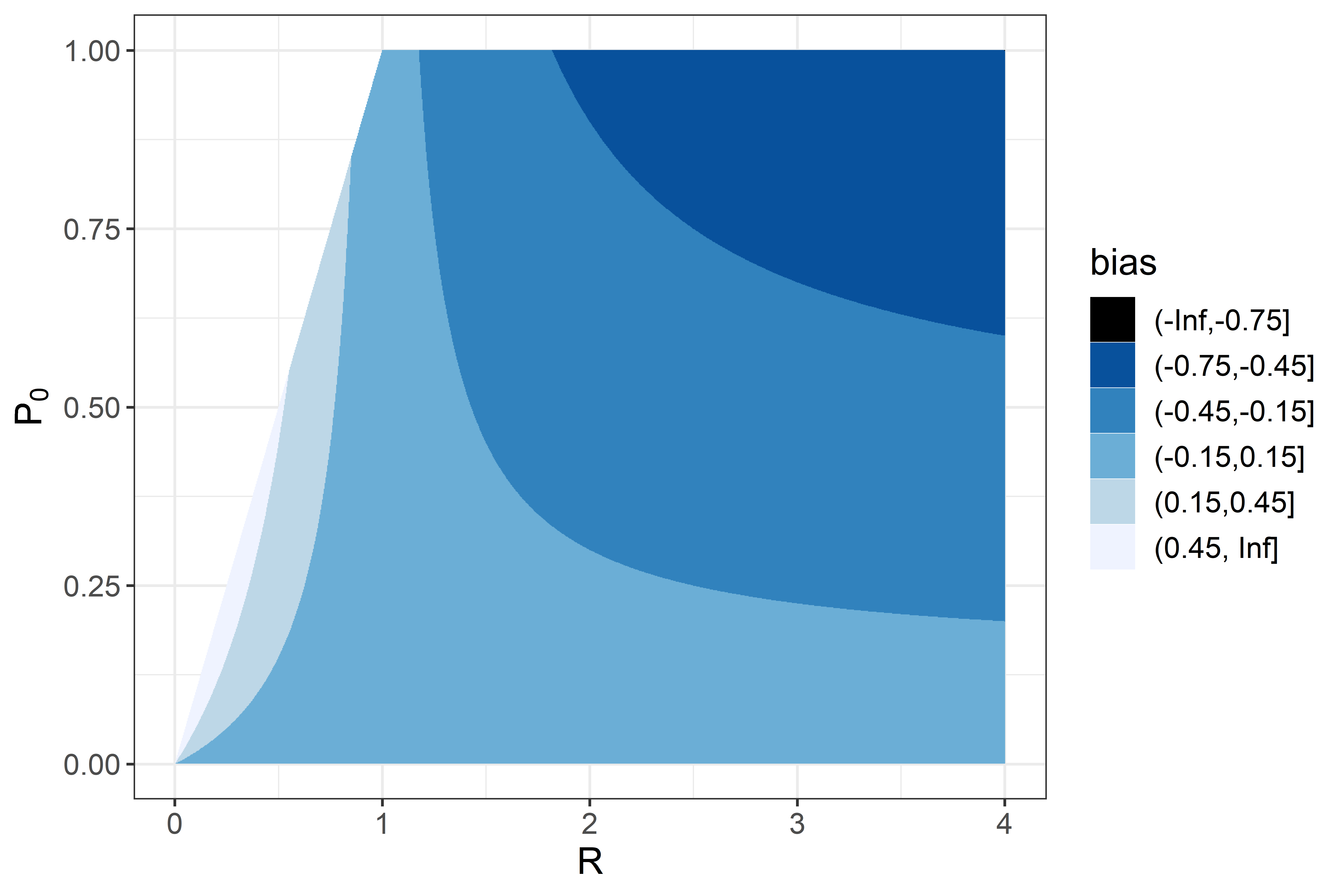

Supplement: Supplemental Information 1 [file peerj-11-15230-s001.png]
