# Supplementary material for: Time series analysis of survival and oviposition cycle duration of Anopheles funestus (Giles) in Mozambique
Source: PeerJ. 2023 May 29;11:e15230. doi: 10.7717/peerj.15230 (PMC10234278; doi:10.7717/peerj.15230)
Supplement: Supplemental Information 2 [file peerj-11-15230-s002.docx]

# Supplementary Information.

# Parous rates in continuously growing or decreasing mosquito population.

When mosquito populations are sampled for a limited period only, rather than over one or more complete annual cycles, the equivalence cannot be assumed between survival and the parous rate. The relationship may better be approximated by that in an exponentially growing mosquito population with constant survival. If there are $T_{t}$ host-seeking mosquitoes at time *t*, some proportion $P_{o}^{(1)}$are parous, and the number of mosquitoes is multiplied at each cycle by a factor $R$*,* then:$\begin{aligned} T_{t} =RT_{t-\theta_{o}} .\#S1 \end{aligned}$

where $\theta_{o}$ is the duration of the full oviposition cycle. If the survival per cycle is $P_{0}$, then the number of parous mosquitoes represent the survivors of the mosquitoes present one cycle earlier. Hence:

$$\begin{aligned} P_{o}^{\left( 1 \right)}T_{t}=P_{0}T_{t-\theta_{o}},\#S2\#\# \end{aligned}$$

Substituting the expression for $T_{t}$ in equation S1 into equation S2, dividing both sides by $T_{t-\theta_{o}}$ and rearranging to obtain an expression for the parous rate gives:

$$\begin{aligned} P_{o}^{\left( 1 \right)}={P_{0}}/R.\#S3 \end{aligned}$$

The bias associated with the use of the parous rate as an estimate of $P_{0}$ is then: $\begin{aligned} P_{o}^{(1)}-P_{0} ={P_{0}}/R-P_{0}.\#S4 \end{aligned}$

Figure S1 is a heatmap showing this bias for a range of values of *R* and $P_{0}.$ The bias is positive for shrinking populations and negative for growing ones, with the largest absolute bias if survival is low and the population falling, or if the growth rate of the population is very high and survival high.

**Figure S1. Bias in parous rate estimates of survival per cycle**


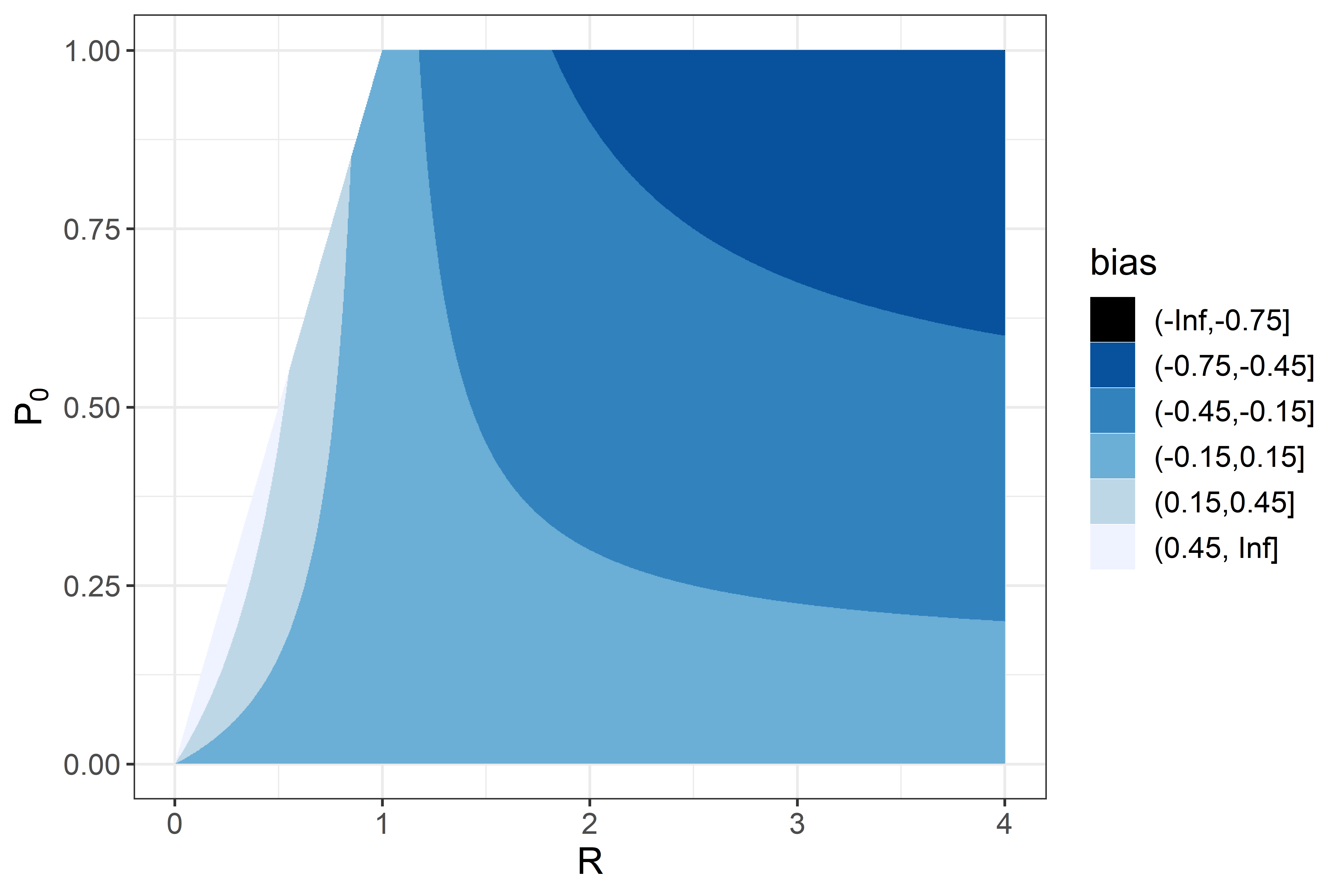


If $R<1$ the mosquito population is decreasing and the survival rate then imposes a limit on the possible rate of decrease, namely $R\geq P_{0}$. The unshaded triangle in the top left of the figure represents the subset of the parameter space that consequently does not arise.
